# From pixels to prognosis: radiomics and AI in Alzheimer’s disease management

**DOI:** 10.3389/fneur.2025.1536463

**Published:** 2025-01-29

**Authors:** Danting Peng, Weiju Huang, Ren Liu, Wenlong Zhong

**Affiliations:** Radiology Department, Chonggang General Hospital, Chongqing, China

**Keywords:** Alzheimer’s disease, AD, radiomics, artificial intelligence, deep learning, neurodegenerative diseases, biomarkers, neuroimaging

## Abstract

Alzheimer’s disease (AD), the leading cause of dementia, poses a growing global health challenge due to an aging population. Early and accurate diagnosis is essential for optimizing treatment and management, yet traditional diagnostic methods often fall short in addressing the complexity of AD pathology. Recent advancements in radiomics and artificial intelligence (AI) offer novel solutions by integrating quantitative imaging features and machine learning algorithms to enhance diagnostic and prognostic precision. This review explores the application of radiomics and AI in AD, focusing on key imaging modalities such as PET and MRI, as well as multimodal approaches combining structural and functional data. We discuss the potential of these technologies to identify disease-specific biomarkers, predict disease progression, and guide personalized interventions. Additionally, the review addresses critical challenges, including data standardization, model interpretability, and the integration of AI into clinical workflows. By highlighting current achievements and identifying future directions, this article underscores the transformative potential of AI-driven radiomics in reshaping AD diagnostics and care.

## 1 Introduction

Alzheimer’s disease (AD) is the most common neurodegenerative disorder and the leading cause of dementia. With the aging global population, the incidence of AD is expected to continue rising, placing increasing pressure on healthcare systems and families (1, 2). Early diagnosis not only helps patients and their families better plan and manage the disease but also allows patients to benefit sooner from available treatments and interventions. Despite advances in medical imaging technologies providing new perspectives for AD diagnosis, traditional imaging analysis methods have limitations when handling complex, multidimensional data. Existing limitations in technology are primarily related to diagnostic and detection models that focus on distinguishing this disease from others with similar symptoms (3, 4). Prognostic models aim to predict the potential progression and outcomes of the disease following diagnosis, including recovery patterns, responses to treatments, and long-term management. However, conventional linear and one-dimensional analytical approaches face challenges in processing complex, multidimensional, and nonlinear data, which restricts their accuracy, generalizability, and overall clinical utility. Additionally, radiologists face enormous workloads and time pressures, increasing the risk of diagnostic errors. Therefore, there is an urgent need to develop new tools and methods to improve diagnostic accuracy and efficiency. The combination of machine learning (ML) and radiomics offers a promising solution to these challenges. ML algorithms can process and analyze large amounts of multidimensional data, while radiomics can extract rich quantitative information from medical images (5, 6). This combination is expected to enhance the diagnostic accuracy of AD, support clinical decision-making, and ultimately improve patient prognosis.This review aims to outline the applications of ML and radiomics in the diagnosis and prognosis of AD and discuss how these technologies can help overcome the limitations of traditional methods. We will explore the latest research advancements and discuss future research directions and potential clinical applications.

## 2 Overview of AD

**Figure 1:**
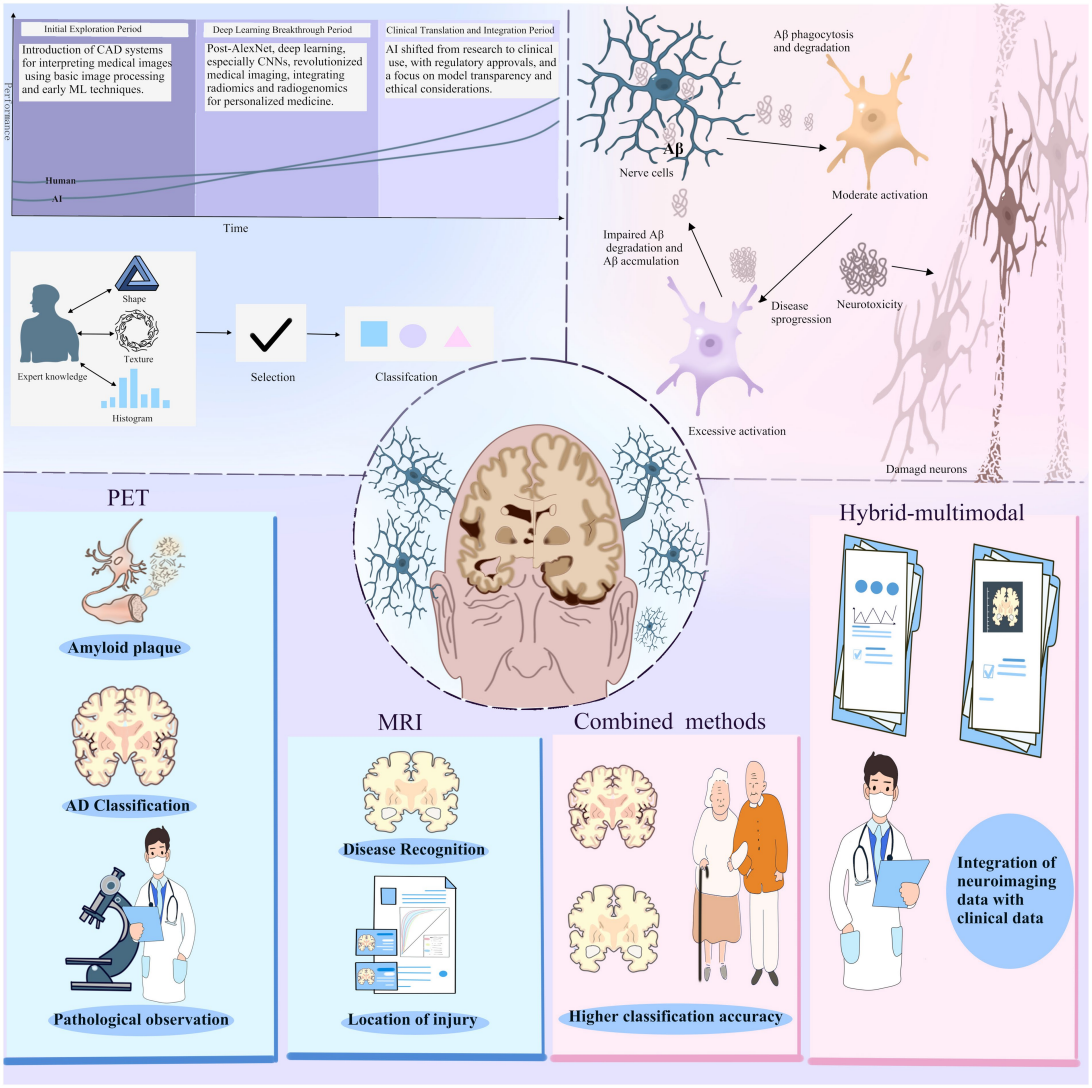
At a time when the field of artificial intelligence is hot, the field of imaging histology has followed the entry of ai technology and produced changes. The application of AI technology in medical image interpretation has gone through three main periods: the initial exploration period, the deep learning breakthrough period, and the clinical translation and integration period. For Alzheimer’s disease, the current result is machine learning combined with PET and MRI images.Positron emission tomography (PET) is used to identify amyloid plaques in the brain, while magnetic resonance imaging (MRI) provides detailed images of the brain structure.The combined use of PET and MRI provides more comprehensive pathological information, which helps in the diagnosis and research of AD. In addition, the hybrid multimodal approach, which integrates neuroimaging data with clinical data and uses AI to process and analyze data from multiple sources, improves classification accuracy.

Alzheimer’s disease is a neurodegenerative disorder affecting the majority of the elderly population, leading to the degeneration of neurons in selected regions (7). The deposition of amyloid plaques and neurofibrillary tangles represents major pathologies in the brain during AD, which, via synaptic dysfunction, results in neuronal loss and a general progressive decline in cognitive capabilities**(Figure** **1****)** (8, 9).

In the initial phase of Alzheimer’s disease (AD), patients often show memory deficits that are linked to the atrophy of both the hippocampus and the entorhinal cortex.As the disease progresses, pathological changes gradually spread to other brain regions, affecting more cognitive functions (10, 11). According to the cognitive decline in AD, it may be divided into several stages: first, the preclinical stage characterized by mild cognitive changes that can seldom be detected using the standard cognitive tests. The next stage is that of mild cognitive impairment, where although patients have a decline in cognitive abilities, it does not usually interfere with daily life; this is followed by the stage of dementia in which there is severe cognitive impairment with marked deterioration in daily functioning (12).

Diagnosis and monitoring could be performed for AD by using different neuroimaging techniques, such as PET, which would detect accumulation of amyloid and tau proteins, and structural MRI, which would show brain atrophy and white matter lesions.Furthermore, laboratory assessments, such as cerebrospinal fluid (CSF) analysis—where a reduction in Aβ levels is observed—and blood biomarkers like neurofilament light chain, offer potential biomarkers that are indicative of AD.These imaging and biomarker data offer important bases for radiomics analysis (13, 14).Currently, predictive models of AD are becoming much finer and more personalized, considering demographic data such as age, sex, and education, and also genetic information, like the presence of the APOEε4 variants, which involves increased risk for AD and thus provides a scientific basis for early intervention and personalized treatment (15).

## 3 Basic concepts of radiomics

Radiomics is an emerging technology in medical imaging analysis that involves extracting numerous quantitative features from various medical imaging modalities, such as CT, MRI, and PET scans, with the aim of identifying patterns and potential biomarkers related to disease. Compared with traditional image analysis, radiomics can process and analyze vast amounts of imaging data, extracting thousands of features. It could potentially be applied across a range of imaging modalities, including both structural techniques, such as CT and MRI, as well as functional methods like positron emission tomography (PET) and single photon emission computed tomography (SPECT).Integration of data coming from several imaging modalities allows achieving a view of the disease more complete for a better diagnosis and accuracy of prediction (16, 17). In the field of neuromedicine, numerous researchers have integrated radiomics with the characteristics of neurological diseases to inform treatment and prognosis. For example, radiogenomic features have been utilized to evaluate the prognosis and treatment response of patients with low-grade glioma (LGG) (18).

## 4 Fundamentals of AI and machine learning

In radiology, trained physicians detect, characterize, and monitor diseases by visually evaluating medical images and reporting findings. This evaluation is typically influenced by factors such as education and experience, and it may occasionally involve subjective judgment (19, 20). In contrast to these more qualitative approaches, artificial intelligence is adept at identifying intricate patterns within imaging data and can offer quantitative assessments in an automated way. When integrated into clinical workflows as a supplementary tool for physicians, AI has the potential to enhance the accuracy and reproducibility of radiological evaluations.

Machine learning (ML) algorithms, a subset of AI, allow systems to learn from structured data and past experiences. By identifying patterns in data, they can also use previously unrecognized trends to make predictions about future human-related data. There are supervised learning (SL), which involves testing against known or labeled dependent variable data, and unsupervised learning (UL), which uses unknown or unlabeled data. Machine learning has long been routinely applied to the screening of biomarkers, as well as the evaluation of prognosis and treatment outcomes for various diseases, including Alzheimer’s disease (AD). Currently, numerous mature machine learning models are available, and interdisciplinary research integrating machine learning with diverse diseases has developed to a highly sophisticated and varied extent (21, 22, 23).

Deep learning (DL), a subset of machine learning (ML), is particularly effective when dealing with large volumes of complex, unstructured data, and it integrates well with radiomics. DL involves multilayer algorithms of artificial neural networks (ANNs), with each algorithm providing interpretations of the data at different hierarchical levels. Deep learning algorithms are capable of learning feature representations from data autonomously, without relying on predefined definitions from human experts. This data-driven method facilitates the creation of more abstract feature definitions, which can enhance both their informativeness and generalizability. As a result, deep learning can assist in quantifying phenotypic characteristics of human tissues while reducing the need for extensive manual preprocessing**(Figure** **1****)** (24).

## 5 Applications of machine learning in Alzheimer’s disease

### 5.1 PET-based radiomics approach

The brain’s glucose metabolism rate is an important indicator of brain activity and neuronal function (25). In AD, decreased glucose metabolism in specific brain regions is closely linked to disease progression. The 18F-FDG-PET (fluorodeoxyglucose-PET) technique can quantify glucose metabolism rates in different brain areas, providing biomarkers for AD classification and prediction (26, 27). Radiomics methods can extract a large number of quantitative features from 18F-FDG-PET images, including texture analysis, shape analysis, and small voxel-based morphological measurements. These features reflect the heterogeneity of brain glucose metabolism and help differentiate AD patients from healthy controls, as well as predict the conversion of mild cognitive impairment (MCI) to AD. For instance, Jiaxuan Peng and his team utilized 18F-FDG-PET radiomic features derived from white matter, combined with machine learning techniques, to effectively predict the progression from MCI to AD. (28).

Amyloid plaques, a hallmark pathological feature of AD, play a crucial role in the disease’s development. Amyloid PET uses tracers such as 11C-PiB, 18F-AV45, 18F-Flutemetamol, and 18F-Florbetaben to directly image amyloid plaques, providing a clear diagnostic approach (29). Radiomics analysis can further extract features from amyloid PET images to enhance diagnostic accuracy (30). According to research by Ying-Hwey Nai and colleagues, three automated methods for classifying amyloid PET images were compared, showing that machine learning (ML) algorithms and deep learning (DL) networks achieved high accuracy and diagnostic confidence in amyloid PET image classification. The research analyzed 276 11C-PiB and 209 18F-AV45 PET scans from the ADNI database and a local cohort, comparing them through global average and maximum SUVR cut-points derived from ROC analysis. The findings suggested that the ML-based classification method achieved comparable accuracy to the ROC classification, but demonstrated superior convergence between training and unseen data, while requiring fewer training samples. Among 68 ML models, the Naive Bayes algorithm performed best overall (31). Amyloid PET has shown good performance in diagnosing AD, particularly in distinguishing AD from normal controls. However, distinguishing AD from MCI patients requires consideration of the potential for MCI patients to progress to AD. Multimodal diagnostic approaches and ML analysis may effectively enhance diagnostic accuracy. Future studies may further optimize ML methods for higher diagnostic precision.

Abnormal phosphorylation and aggregation of tau protein, leading to neurofibrillary tangles, is another key pathological feature of AD. Tau PET uses tracers such as 18F-flortaucipir to directly image the distribution and accumulation of tau protein, providing new insights into the disease’s pathological changes. Radiomics methods can extract detailed features from tau PET images, including the heterogeneity and distribution patterns of tau deposits. Park and his team developed a 3D-CNN deep learning network designed to classify AD, MCI, and healthy controls using tau PET images, highlighting its capability in distinguishing various stages of AD (32). Moreover, Gebre and colleagues introduced an advanced tau summary measure that quantifies tau deposition heterogeneity into a single number, called the Tau Heterogeneity Evaluation in Alzheimer’s Disease (THETA). This model uses region-specific SUV ratios and normalizes them against the uptake in the cerebellar cortex. Using explainable AI methods (Shapley additive explanation), the researchers estimated feature contributions and formulated a global tau summary measure for each participant. The THETA method achieved 95% balanced accuracy on the Mayo test set, with an accuracy of at least 87% on the validation set, highlighting its significant potential for accurately identifying tau deposition in clinical applications (33).

### 5.2 MRI-based radiomics approach

MRI data is also commonly used in radiomics analysis. A review by Avinash Chandra and colleagues emphasized the crucial role of MRI technology in distinguishing AD from MCI. MRI can identify brain damage patterns that distinguish AD from other neurological conditions and also highlight risk factors linked to the progression from MCI to AD (34). Deep learning (DL) is particularly useful when dealing with large and complex unstructured data. Shangran Qiu and his team developed a deep learning framework that integrates fully convolutional networks (FCN) with multilayer perceptrons (MLP) to generate high-resolution disease probability maps from MRI images. This framework can generate precise and intuitive individual AD risk assessments, enabling accurate diagnosis. The model was trained on the ADNI dataset and validated on independent cohorts from the Australian Imaging, Biomarker, and Lifestyle Flagship Study (AIBL), the Framingham Heart Study (FHS), and the National Alzheimer’s Coordinating Center (NACC). The model demonstrated excellent performance, maintaining consistent accuracy across datasets and outperforming diagnostic performance of practicing neurologists (35). MRI techniques, such as structural MRI, diffusion tensor imaging (DTI), arterial spin labeling (ASL), magnetic resonance spectroscopy (MRS), and functional MRI (fMRI), have shown potential in differentiating AD from MCI. However, at present, only traditional structural imaging is typically recommended for routine clinical application. Encouragingly, Samuel L. Warren and colleagues explored the use of functional MRI (fMRI) combined with deep learning techniques, particularly deep neural networks (DNNs), in AD diagnosis. fMRI can detect brain patterns in participants’ scans, and deep learning models can denoise these images to improve diagnostic accuracy. However, the study also pointed out limitations such as the need for large data sets, model interpretability issues, and generalization across different populations and datasets (36).

### 5.3 Combined neuroimaging approaches

Research integrating MRI and PET has demonstrated improved classification accuracy over studies that rely on a single neuroimaging modality (37). By using both MRI and PET data for training, more comprehensive information and higher predictive accuracy can be obtained for neuroimaging analysis. The combined MRI and PET dataset takes advantage of the complementary strengths of both modalities in structural and functional imaging: MRI provides clear anatomical structures and tissue characteristics, while PET reveals micro-dynamics of brain metabolic activities. When both data types are used in training, the model can capture both anatomical abnormalities and changes in metabolism and function, significantly enhancing disease detection sensitivity and specificity.

In practical applications, combining MRI and PET allows clinicians and researchers to obtain both structural and metabolic information in a single imaging process. For neurodegenerative diseases like AD, PET/MRI combined imaging can detect metabolic abnormalities while observing anatomical changes, helping identify early metabolic shifts in the disease and providing a basis for timely intervention and precise diagnosis. Deep learning models, such as convolutional neural networks (CNNs) and autoencoders, can automatically extract richer features from multimodal data, learning potential associations between the high-resolution structural information from MRI and the functional activity patterns from PET. This is particularly useful in the early detection and progression evaluation of AD (38, 39).

Furthermore, data fusion strategies (e.g., feature-level fusion and decision-level fusion) can integrate MRI and PET information into a unified framework to construct a joint feature space, enhancing the model’s generalization capability and predictive accuracy (40, 41).

### 5.4 Hybrid multimodal approaches

Recent research has investigated the integration of neuroimaging data with clinical information, such as CSF biomarkers, blood tests, and cognitive assessments, as a potential strategy for AD diagnosis. Research by Xianfeng Yu and colleagues demonstrated an innovative multivariate predictive model that integrates MRI radiomic features and plasma biomarkers to improve AD prediction accuracy. The study utilized two independent cohorts: the Sino Longitudinal Study on Cognitive Decline (SILCODE) and the Alzheimer Disease Neuroimaging Initiative (ADNI). Data collected included comprehensive assessments, MRI scans, plasma samples, and amyloid PET images. A new composite index was developed through multivariate logistic regression, incorporating both plasma and MRI radiomic biomarkers. The model’s performance and generalization ability were evaluated across two racially diverse cohorts, demonstrating the potential of multivariate models combining MRI radiomics and plasma biomarkers in predicting AD conversion, offering new perspectives for early diagnosis and treatment of AD (42). Integrating CSF biomarkers (e.g., Aβ42, p-Tau, t-Tau), blood biomarkers (e.g., neurofilament light chain, glial fibrillary acidic protein), and cognitive assessments (e.g., the Mini-Mental State Examination, MMSE) may offer valuable insights into the progression of AD. These biomarkers reflect molecular and cellular changes in the brain and aid in identifying the disease before symptoms appear.

## 6 Challenges and future directions

### 6.1 Data-related challenges

#### 6.1.1 Foundations of reliable Radiomics

The optimal sample size is influenced by several factors, such as image quality, the algorithms used, and the desired predictive outcomes. In practice, especially for neuroimaging analysis, sample sizes are particularly hard to get. Small sample sizes most often result in overfitting of the model to the training data. This means the model performs well on the training set but generalizes poorly to new, unseen data. Overfitting overestimates the true performance of the model (43, 44). Andrius Vabalas et al. (43) and Robyn Larracy et al. (45) have suggested further that new strategies in collecting and sharing of data are developed in future, with a view to improve available sample size. The use of cross-validation and ensemble learning is also recommended to provide additional robustness and generalization where small sample size models are developed.

The radiomics workflow introduces significant variability, which may contribute to challenges in reproducibility and result in inconsistent findings. Different studies may use varying imaging parameters and preprocessing steps, such as image denoising, normalization, and segmentation, resulting in differences in data quality and feature extraction (46, 47). Radiomics analysis involves extracting a large number of features from imaging data, and different studies might select different feature sets, affecting model comparison and integration (48, 49). The reporting of study results may lack uniform standards, making it difficult for other researchers to understand and replicate findings (50). As a result, there is currently no standardized protocol for ML-based radiomics analysis in neuroimaging. Future studies may aim to establish best practices and standardized workflows to improve consistency and reliability in this area (51).

#### 6.1.2 Harmonizing various imaging data for comprehensive analysis

Integrating structural MRI with functional PET data may enhance diagnostic performance. However, challenges remain in ensuring the generalizability of these combined models and avoiding overfitting as dimensionality increases (52). Future research needs to focus on developing and implementing standardized processes, data dimensionality reduction techniques, effective model training and validation methods, and stringent quality control measures to overcome these challenges. For instance, statistical methods and machine learning techniques can be used to select the most predictive features, reducing model complexity. Methods such as Principal Component Analysis (PCA) and t-distributed Stochastic Neighbor Embedding (t-SNE) are useful for reducing data dimensionality while preserving key information (53). Applying L1 (LASSO) or L2 (ridge regression) regularization can limit model complexity and enhance generalization capabilities (54, 55).

### 6.2 Balancing model complexity and clinical practicality

Concerns have also been raised about the interpretability of current ML models. In many studies, models are selected through multiple comparisons to assess the predictive accuracy of various algorithms, often prioritizing the most accurate model. In the study by Sucheta Chauhan and colleagues, four different methods were compared: Convolutional Neural Networks (CNN), Ridge Regression (RR), PCA-based RR (PCA + RR), and a hybrid model combining higher-order CNN features with PCA + RR. The predictive accuracy of these methods was evaluated through multiple comparisons. Although CNN models exhibited higher predictive accuracy in some cases, they are often considered “black box” models because their decision-making processes are opaque and difficult to interpret. This leads to a lack of understanding of why the models perform well and what key features they identify. The lack of transparency not only affects the comprehension of model performance but also impacts the medical interpretation of predictive results. In clinical applications, understanding why a model makes a specific prediction is crucial, as it is essential for the model’s acceptance and practical use (56).

Amid the current boom in artificial intelligence, the field of radiomics is undergoing transformation with the integration of AI technology. This is not merely an upgrade in equipment technology and diagnostic capabilities; broadly speaking, it is a revolution from labor (radiologists) to medical productivity. From a labor perspective, the combination of deep learning and multiple diagnostic platforms requires primary diagnosing physicians to have certain statistical literacy, especially radiologists. During the incomplete phase of deep learning and radiomics development, they need to apply models with different focuses and capabilities to various patient situations to maximize diagnostic performance. From a productivity standpoint, with the help of AI, the efficiency of a highly trained radiologist far exceeds that of multiple traditional high-level radiology technicians. This does not imply a reduction in future radiologist positions; rather, it necessitates more high-level radiologists proficient in new technologies and AI multidisciplinary researchers. In an ideal scenario where technological advancement in AI and labor structure reform complement each other, it is possible to achieve productivity far exceeding traditional levels with the same number of personnel.
